# Tumor volume and tumor crossing of the axial renal midline predict renal function after robotic partial nephrectomy

**DOI:** 10.1038/s41598-021-01539-1

**Published:** 2021-11-18

**Authors:** Haruyuki Ohsugi, Kyojiro Akiyama, Hisanori Taniguchi, Masaaki Yanishi, Motohiko Sugi, Tadashi Matsuda, Hidefumi Kinoshita

**Affiliations:** Department of Urology and Andrology, Kansai Medical University, 2-3-1 Shin-machi, Hirakata, Osaka 573-1191 Japan

**Keywords:** Oncology, Risk factors, Urology

## Abstract

There are several nephrometry scoring systems for predicting surgical complexity and potential perioperative morbidity. The R.E.N.A.L. scoring system, one of the most well-known nephrometry scoring systems, emphasizes the features on which it is based (Radius, Exophytic/endophytic, Nearness to collecting system or sinus, Anterior/posterior, and Location relative to polar lines). The ability of these nephrometry scoring systems to predict loss of renal function after robotic partial nephrectomy (RPN) remains controversial. Therefore, we verified which combination of factors from nephrometry scoring systems, including tumor volume, was the most significant predictor of postoperative renal function. Patients who underwent RPN for cT1 renal tumors in our hospital were reviewed retrospectively (n = 163). The preoperative clinical data (estimated glomerular filtration rate [eGFR], comorbidities, and nephrometry scoring systems including R.E.N.A.L.) and perioperative outcomes were evaluated. We also calculated the tumor volume using the equation applied to an ellipsoid by three-dimensional computed tomography. The primary outcome was reduced eGFR, which was defined as an eGFR reduction of ≥ 20% from baseline to 6 months after RPN. Multivariable logistic regression analyses were used to evaluate the relationships between preoperative variables and reduced eGFR. Of 163 patients, 24 (14.7%) had reduced eGFR. Multivariable analyses indicated that tumor volume (cutoff value ≥ 14.11 cm^3^, indicating a sphere with a diameter ≥ approximately 3 cm) and tumor crossing of the axial renal midline were independent factors associated with a reduced eGFR (odds ratio [OR] 4.57; 95% confidence interval [CI] 1.69–12.30; P = 0.003 and OR 3.50; 95% CI 1.30–9.46; P = 0.034, respectively). Our classification system using these two factors showed a higher area under the receiver operating characteristic curve (AUC) than previous nephrometry scoring systems (AUC = 0.786 vs. 0.653–0.719), and it may provide preoperative information for counseling patients about renal function after RPN.

## Introduction

Partial nephrectomy (PN) is the current standard treatment for the management of small renal tumors to reduce the risk of developing postoperative chronic kidney disease (CKD)^1^. In particular, a warm ischemic time (WIT) of > 25 min is associated with short- and long-term renal consequences^2^. Recent meta-analyses have shown that the WIT is significantly shorter in robotic partial nephrectomy (RPN) than in laparoscopic partial nephrectomy^3^. However, WIT may play only a role in the functional outcome of partial nephrectomy^4^, and preoperative factors other than WIT should be evaluated for predicting postoperative renal function in RPN.

There are some nephrometry scoring systems for predicting surgical complexity and potential perioperative morbidity. The R.E.N.A.L. score emphasizes the features on which it is based (Radius, Exophytic/endophytic, Nearness to collecting system or sinus, Anterior/posterior, and Location relative to polar lines)^5^. The preoperative aspects and dimensions used for anatomic (PADUA) classification includes parameters such as the longitudinal location, exophytic rate, renal rim, renal sinus, urinary collecting system, and tumor size^6^. The diameter-axial-polar (DAP) score is a modified version of the R.E.N.A.L. classification and the centrality index (c-index) and contains tumor diameter scoring, axial distance scoring, and polar distance scoring^7,8^. Whether these nephrometry scoring systems have the ability to predict loss of renal function after PN is still controversial^9,10^. Furthermore, some reports that used mathematically calculated scores determined from preoperative images, such as the centrality index (c-index) and tumor contact surface area, predicted that the estimated glomerular filtration rate (eGFR) decreases after PN^8,11^. In addition, tumor volume is more representative of tumor burden than tumor distance and might be correlated with renal function after partial nephrectomy^12,13^. However, to our knowledge, the combination of nephrometry scoring systems and tumor volume has not been evaluated for predicting renal function after RPN.

Therefore, we revealed factors, using previous nephrometry scoring systems with the addition of tumor volume, that are associated with reduced postoperative renal function and used this combination of factors to create a new scoring system that predicts the reduction in postoperative renal function. Finally, we compared the accuracy of the new classification system with R.E.N.A.L., PADUA, and DAP scores for predicting eGFR reduction.

## Results

### Patient characteristics

The clinical patient characteristics are shown in Table 1. The median percent decrease in eGFR 6 months after RPN was 7.55% (interquartile range [IQR]: 1.42–15.29%). Of 163 patients, 24 (14.7%) had an eGFR reduction of ≥ 20% from baseline to 6 months after RPN. The median tumor volume was 6.28 cm^3^ (IQR: 2.70–14.68 cm^3^). The nephrometry scores (R.E.N.A.L. and DAP scores) are shown in Table 2. The median R.E.N.A.L. score was six, and the median DAP score was five. No postoperative complications greater than Grade 3 in Clavien–Dindo Classification were observed.

**Table 1:** Clinical patient characteristics.

Variables	
N	163
Age (years), median (IQR)	66 (56–73)
**Sex, n (%)**	
Female	50 (30.7)
Male	113 (69.3)
BMI (kg/m^2^), median (IQR)	23.6 (21.9–25.6)
**ASA, n (%)**	
1	34 (20.9)
2	117 (71.8)
3	12 (7.4)
Preoperative SCr (mg/dl), median (IQR)	0.80 (0.67–0.98)
Preoperative eGFR (ml/min/1.73 m^2^), median (IQR)	70.0 (57.0–82.5)
Preoperative eGFR < 60, n (%)	47 (28.8)
Comorbidity of DM	30 (18.4)
Comorbidity of HTN	75 (46.0)
Previous abdominal surgery	52 (31.9)
Antiplatelet of anticoagulant therapy	24 (14.7)
Tumor diameter (cm), median (IQR)	2.5 (1.95–3.35)
Tumor volume (cm^3^), median (IQR)	6.28 (2.70–14.68)
Distance from the tumor to the collecting system (mm), median (IQR)	14 (8 - 22)
**Approach, n (%)**	
Intraperitoneal	99 (60.7)
Retroperitoneal	64 (39.3)
**Surgical side, n (%)**	
Right	87 (53.4)
Left	76 (46.6)
**Pathological subtype, n (%)**	
Clear cell RCC	119 (73.0)
Chromophobe RCC	14 (8.6)
Papillary RCC	9 (5.5)
Clear cell papillary RCC	4 (2.5)
Benign neoplasm (AML or Oncocytoma)	9 (5.5)
Others	8 (5.0)
**Pathological T stage, n (%)**	
pT1a	121 (74.2)
pT1b	12 (7.4)
pT2	0 (0.0)
pT3a	20 (12.3)
Indeterminable	10 (6.1)
WIT (s), median (IQR)	1149 (883–1456)
Estimated blood loss (ml), median (IQR)	100 (31–200)
Postoperative eGFR decrease (%), median (IQR)	7.55 (1.42–15.29)
Postoperative eGFR decrease greater than 20%, n (%)	24 (14.7)

*IQR* interquartile range, *BMI* body mass index, *ASA* American Society of Anesthesiologists, *SCr* serum creatinine, *eGFR* estimated glomerular filtration rate, *DM* diabetes mellitus, *HTN* hypertension, *RCC* renal cell carcinoma, *AML* angiomyolipoma, *WIT* warm ischemia time.

**Table 2:** Nephrometry scores.

Variables	
N, (%)	163
R.E.N.A.L. score, median (IQR)	6 (5–7)
**R**	
1	142 (87.1)
2	21 (12.9)
**E**	
1	58 (35.6)
2	88 (54.0)
3	17 (10.4)
**N**	
1	118 (72.4)
2	19 (11.7)
3	26 (16.0)
**A**	
x	28 (17.2)
a	60 (36.8)
p	75 (46.0)
**L**	
1	72 (44.2)
2	50 (30.7)
3	41 (25.2)
**R.E.N.A.L. score risk categorization, n (%)**	
Low (4–6)	100 (61.3)
Intermediate (7–9)	52 (31.9)
High (≥ 10)	11 (6.7)
DAP score, median (IQR)	5 (4–6)
**Diameter**	
1	72 (44.2)
2	81 (49.7)
3	10 ( 6.1)
**Axial**	
1	61 (37.4)
2	69 (42.3)
3	33 (20.2)
**Polar**	
1	67 (41.1)
2	55 (33.7)
3	41 (25.2)

*R.E.N.A.L.* Radius, exophytic/endophytic, nearness to collecting system or sinus, anterior/posterior, and location relative to polar lines; *DAP* diameter-axial-polar; *IQR* interquartile range.

### Association between reduced eGFR and each factor of the nephrometry scoring systems including the tumor volume

**Figure 1:**
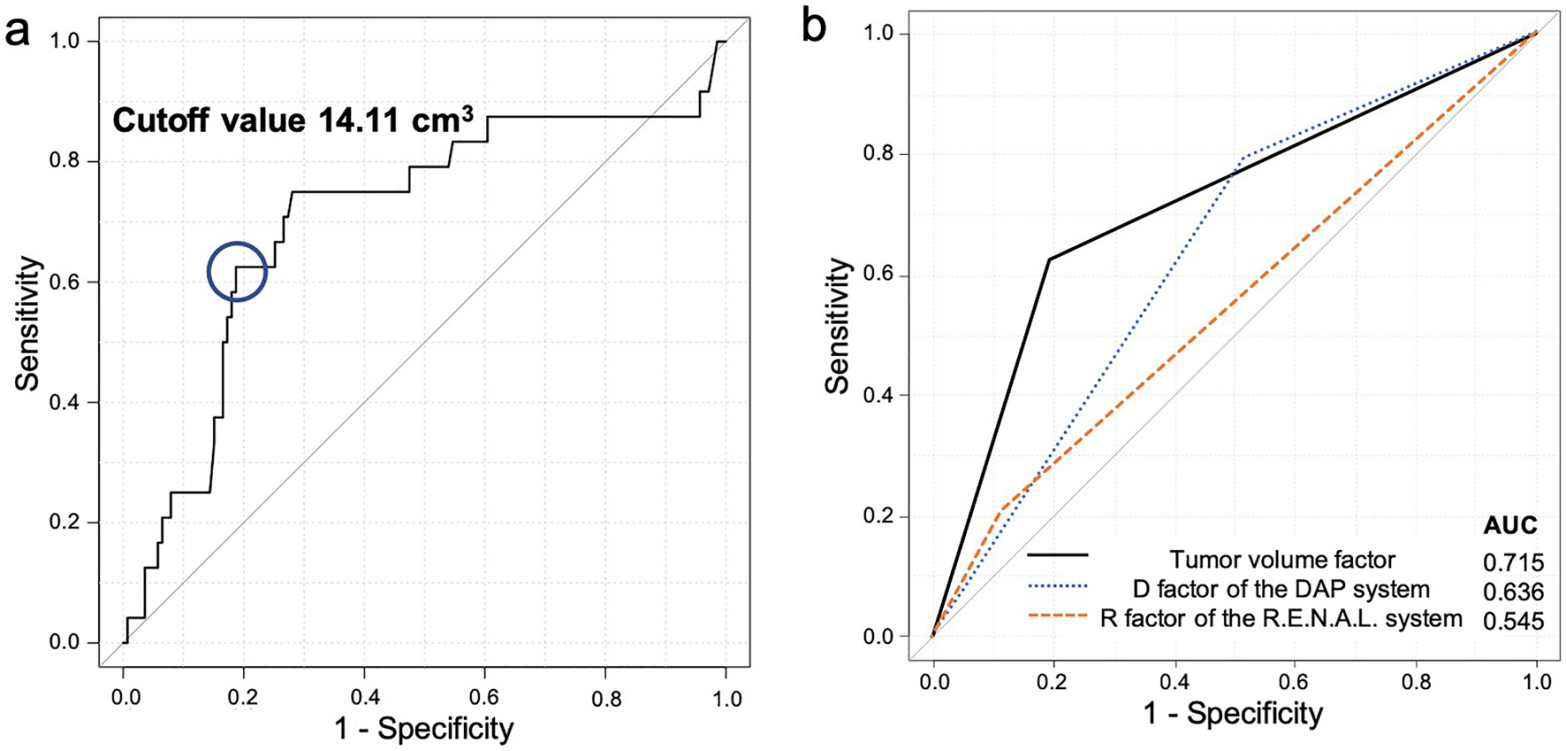
(**a**) Receiver operating characteristic curve of tumor volume and cutoff value for predicting eGFR reduction of ≥ 20%. (**b**) Comparison of the AUC values of the tumor volume (cutoff value ≥ 14.11 cm^3^), D factor of the DAP system, and R factor of the R.E.N.A.L. system for predicting eGFR reduction of ≥ 20%. *AUC* area under the receiver operating characteristic curve; *DAP* diameter-axial-polar; *R.E.N.A.L.* radius, exophytic/endophytic, nearness to collecting system or sinus, anterior/posterior, and location relative to polar lines.

The appropriate cutoff value of tumor volume for predicting reduced eGFR was 14.11 cm^3^ (sensitivity = 0.625 and specificity = 0.806) (Fig. 1a). The tumor volume factor (cutoff value ≥ 14.11 cm^3^, indicating a sphere with a diameter ≥ approximately 3 cm) showed a higher AUC than the size factor of the DAP and R.E.N.A.L. classification systems (0.715 vs 0.547–0.636; Fig. 1b). Hence, the tumor volume factor was considered to be representative of tumor size. Bivariate analyses of each factor of the nephrometry scoring systems are shown in Table 3. In addition to the tumor size factor, the N and L factors of the R.E.N.A.L. system, the P factor of the DAP system, and size factors were found to be significantly associated with the outcome of interest (all P < 0.05). Three points for both the L factor of the R.E.N.A.L. system and the P factor of the DAP system were significant factors for predicting a reduced eGFR. The number of patients with 3 points for the L factor of the R.E.N.A.L. system was the same as the number of patients with 3 points for the P factor of the DAP system (n = 41). Therefore, tumor crossing of the axial renal midline was adopted as an important representation of tumor location relative to polar lines. In terms of the nearness of the tumor to the collecting system, 4–7 mm and ≤ 4 mm were more significant factors for predicting reduced eGFR compared to ≥ 7 mm. Therefore, nearness of the tumor to the collecting system of < 7 mm, which meant 2 or 3 points in the N factor of the R.E.N.A.L. system, was adopted as an important representation of nearness to the collecting system.

**Table 3:** Bivariate analysis of each factor of nephrometry scores for predicting postoperative eGFR decrease.

Variable	Bivariate analysis	
	OR (95% CI)	p value
R.E.N.A.L.-R (1 vs. 2)	2.02 (0.66–6.17)	0.215
R.E.N.A.L.-E (1 vs. 2)	2.54 (0.88–7.32)	0.085
R.E.N.A.L.-E (1 vs. 3)	1.41 (0.25–8.03)	0.696
R.E.N.A.L.-N (1 vs. 2)	3.47 (1.05–11.50)	0.041
R.E.N.A.L.-N (1 vs. 3)	4.32 (1.53–12.20)	0.006
R.E.N.A.L.-A (x vs. a)	0.56 (0.18–1.82)	0.338
R.E.N.A.L.-A (x vs. p)	0.56 (0.18–1.73)	0.317
R.E.N.A.L.-L (1 vs. 2)	0.59 (0.15–2.41)	0.465
R.E.N.A.L.-L (1 vs. 3)	4.81 (1.75–13.20)	0.002
DAP-diameter (1 vs. 2)	3.56 (1.24–10.20)	0.018
DAP-diameter (1 vs. 3)	3.35 (0.56–20.20)	0.187
DAP-axial (1 vs. 2)	1.74 (0.60–5.02)	0.307
DAP-axial (1 vs. 3)	2.47 (0.75–8.08)	0.135
DAP-polar (1 vs. 2)	1.24 (0.34–4.52)	0.745
DAP-polar (1 vs. 3)	6.43 (2.10–19.6)	0.001

*R.E.N.A.L.* Radius, exophytic/endophytic, nearness to collecting system or sinus, anterior/posterior, and location relative to polar lines; *DAP* Diameter-Axial-Polar; *OR* Odds ratio; *CI* confidence interval.

R denotes the maximal diameter of the tumor, with 1 point for a tumor size ≤ 4 cm and 2 points for a tumor size 4–7 cm. E represents the exophytic or endophytic properties of the tumor, with 1 point for ≥ 50% exophytic, 2 points for < 50% exophytic, and 3 points for entirely endophytic. N denotes the nearness of the tumor to the collecting system, with 1 point for ≥ 7 mm, 2 points for 4–7 mm, and 3 points for ≤ 4 mm. A represents whether the tumor is located anterior (a) or posterior (p) to the kidney midline plane. When the tumor grows from the renal poles or arises from the kidney so that a meaningful anterior or posterior designation is not possible, (x) is assigned. L denotes the location relative to the polar lines, with 1 point indicating that the lesion is above the upper or below the lower polar line, 2 points indicating that the lesion crosses the polar line, and 3 points indicating that > 50% of the mass is across the polar line, the tumor crosses the axial renal midline, or the mass is entirely between the polar lines. Diameter represents the maximal diameter of the tumor, with 1 point for a tumor size < 2.4 cm, 2 points for ≥ 2.4–4 cm, and 3 points for > 4 cm. Axial denotes the axial distance from the center point to the closest tumor edge, with 1 point for > 1.5 cm, 2 points for ≤ 1.5 cm, and 3 points for the tumor touching or overlapping the center point. Polar represents the polar distance from the middle plane to the closest tumor edge, with 1 point for > 2 cm, 2 points for ≤ 2 cm, and 3 points for the tumor crossing the axial renal midline.

### Bivariate and multivariable analyses predicting reduced eGFR

Those significant nephrometry factors including tumor volume factor (cutoff value ≥ 14.11 cm^3^) and preoperative clinical patient characteristics were investigated on bivariate and multivariable analyses. The bivariate analyses showed that a comorbidity of DM, tumor volume, nearness of the tumor to the collecting system, and tumor crossing of the axial renal midline were significantly associated with reduced eGFR (all P < 0.05, Table 4). The multivariable analysis showed that tumor crossing of the axial renal midline (OR 3.50; 95% CI 1.30–9.46; P = 0.014) and tumor volume (OR 4.57; 95% CI 1.69–12.30; P = 0.003) were significant independent factors for predicting reduced eGFR (Table 4).

**Table 4:** Bivariate and multivariable analyses of preoperative clinical factors for predicting postoperative eGFR reduction.

Variables	Bivariate analysis		Multivariable analysis^a^	
	OR (95% CI)	p value	OR (95% CI)	p value
Age, years (< 75 vs. ≥ 75)	0.48 (0.13–1.71)	0.255	–	–
Sex (female vs. male)	1.39 (0.52–3.74)	0.515	–	–
BMI, kg/m^2^ (< 25 vs. ≥ 25)	1.25 (0.51–3.08)	0.623	–	–
Preoperative eGFR, ml/min/1.73 m^2^ (≥ 60 vs. < 60)	1.02 (0.39–2.65)	0.969	–	–
DM (absent vs. present)	2.66 (1.01–6.97)	0.047	–	–
HTN (absent vs. present)	1.21 (0.51–2.87)	0.672	–	–
Antiplatelet of anticoagulant therapy (absent vs. present)	0.48 (0.11–2.21)	0.348	–	–
Previous abdominal surgery (absent vs. present)	0.67 (0.25–1.81)	0.434	–	–
N factor in the R.E.N.A.L. system (1 vs. 2/3)/nearness from the tumor to the collecting system, mm (≥ 7 vs. < 7)	3.95 (1.61–9.67)	0.003	–	–
P factor in the DAP system (1/2 vs. 3)/tumor crossing of the axial renal midline (absent vs. present)	5.81 (2.33–14.50)	< 0.001	3.50 (1.30–9.46)	0.014
Tumor volume, cm^3^ (< 14.11 vs. ≥ 14.11)	6.91 (2.74–17.50)	< 0.001	4.57 (1.69–12.30)	0.003

*BMI* body mass index; *eGFR* estimated glomerular filtration rate; *DM* diabetes mellitus; *HTN* hypertension; *R.E.N.A.L.* Radius, exophytic/endophytic, nearness to collecting system or sinus, anterior/posterior, and location relative to polar lines; *DAP* diameter-axial-polar; *OR* odds ratio; *CI* confidence interval.

^a^Backward step-down selection was used.

### Accuracy of our classification system for predicting decreased renal function

**Figure 2:**
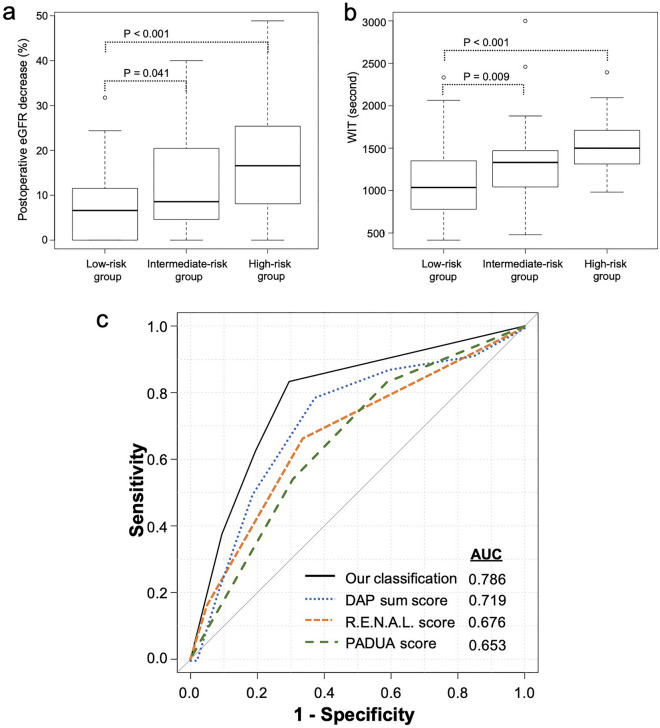
(**a**) Postoperative eGFR decrease (continuous variable) with our classification system. (**b**) Intraoperative WIT with our classification system. (**c**) Comparison of the AUCs of our classification system, the DAP sum score, the R.E.N.A.L. score, and the PADUA score for predicting eGFR reduction of ≥ 20%. *AUC* area under the receiver operating characteristic curve; *DAP* diameter-axial-polar, *eGFR* estimated glomerular filtration rate; *PADUA* preoperative aspects and dimensions used for anatomic; *R.E.N.A.L.* radius, exophytic/endophytic, nearness to collecting system or sinus, anterior/posterior, and location relative to polar lines, *WIT* warm ischemic time.

We developed the new classification system using independent factors including tumor crossing of the axial renal midline and tumor volume (Supplementary Table 1). According to the classification system, all patients were stratified into the following three groups: low-risk group (0 factors, n = 102), intermediate-risk group (1 factor, n = 39), and high-risk group (2 factors, n = 22). The classification system showed a statistically significant trend for predicting postoperative decreases in eGFR (continuous variable) 6 months after RPN (P < 0.001; Fig. 2a) and for predicting the WIT (P < 0.001; Fig. 2b). To ascertain whether our classification system was useful for predicting postoperative reduced eGFR, we compared the predictive accuracy between our classification system and nephrometry scoring systems such as the R.E.N.A.L. score (low, intermediate, and high), PADUA score (low, intermediate, and high), and DAP sum score. Our classification system showed a higher AUC than these nephrometry scoring systems (0.786 vs. 0.653–0.719) in our cohort (Fig. 2c).

## Discussion

The current study demonstrated that tumor volume (cutoff value ≥ 14.11 cm^3^, indicating a sphere with a diameter ≥ approximately 3 cm) and tumor crossing the axial renal midline, were significant factors for predicting eGFR reduction after RPN. The simple classification system using these two factors had the best accuracy for predicting eGFR reduction after RPN compared with existing nephrometry scoring systems such as the R.E.N.A.L., DAP, and PADUA scores. Our classification system can provide prognostic information for counseling patients about renal function after RPN and assist in preoperative decision making.

To assess overall survival benefits in patients with renal cell carcinoma (RCC) after partial or radical nephrectomy, predicting both oncologic outcomes and decreased renal function to avoid chronic kidney disease is essential^14,15^. Preoperative renal function should be evaluated sufficiently before surgery. In addition, analysis of eGFR reduction from baseline to a point after surgery is crucial for accurately predicting postoperative renal function. General functional reduction after PN averages approximately 10% in the two-kidney and 20% in the one-kidney model^16^. A previous report set both 10% and 20% reduction of eGFR from baseline as the cutoff values^11^. In our study, we set the cutoff point for reduced eGFR to 20%, because it was close to the definition of acute kidney injury, which is a > 25% reduction in eGFR from baseline, and was considered clinically more significant^17^.

Recent reports have shown that nomograms that accurately predict eGFR reduction after PN incorporated the R.E.N.A.L. score in addition to sex, age, and preoperative renal function^17,18^. Therefore, the nephrometry scoring systems might be able to predict a decline in renal function after PN. Simmons et al. reported that the DAP score, which is a modified version of the R.E.N.A.L. classification and c-index, is associated with volume loss and renal function after PN^7^. The DAP score includes three different variables including the tumor diameter, axial distance from the center point, and polar distance from the midline. Interestingly, the three factors, including tumor volume, nearness to the collecting system (< 7 mm), and tumor crossing of the axial renal midline, which were significantly associated with eGFR reduction after RPN in our study, were similar to each of the DAP. Among these three factors, nearness to the collecting system was not an independent factor associated with the outcome of interest in our study. However, the distance from the tumor to the collecting system tended to be shorter as the score of P factor in the DAP increased (Supplementary Fig. 2a, p < 0.001). Moreover, it was negatively correlated with tumor volume (Supplementary Fig. 2b, R = 0.481, p < 0.001). Thus, the two independent factors used in our classification system may also reflect proximity to the collecting system.

In complex cases, long ischemia times are required for complete tumor resection^19^. For renal function preservation, various techniques during PN have been described (e.g., off-clamp, selective/super-selective clamp, and early unclamp, or cooling techniques for hypothermia)^20,21^. We showed that our classification system was significantly correlated with the WIT (Fig. 2b); thus, our classification system might be related to the complexity of the surgery. Therefore, our classification system might help to select patients who need various surgical techniques to avoid renal insufficiency.

Our results should be interpreted with caution because of several limitations. First, this study was based on data from patients who were treated at a single center. Second, external or internal validations are needed before applying the classification system for selecting patients, but these have not been performed. Third, the study was retrospective in design with, and the follow-up period was relatively short. Fourth, the patient population in our study entirely comprised patients who underwent RPN with localized cT1 renal tumors. Watts et al. reported that R and E in the R.E.N.A.L. classification were associated with postoperative renal function of the surgical kidney^22^. The differences may be due to the criteria used for patient selection, such as surgical approach or tumor size. Our results should be fitted in patients who underwent RPN with cT1 tumors. Fifth, while perioperative variables such as the WIT and estimated blood loss were not considered, these variables are likely important influencers of postoperative renal function. However, the purpose of this study was to determine which combination of preoperative factors such as nephrometry scoring systems were best for predicting eGFR reduction. We also calculated the tumor volume assuming that each tumor was an ellipsoid. This was not a true volume, but the calculation of tumor volume is easily obtained from preoperative three-dimensional computed tomography (3D-CT) scans.

## Conclusion

Tumor volume and tumor crossing of the axial renal midline were independent predictors of eGFR reduction after RPN. Our classification system using these two factors had the best accuracy for predicting postoperative eGFR reduction when compared with previous nephrometry scoring systems such as the R.E.N.A.L., DAP, and PADUA scores.

## Methods

### Patient selection

The medical records of 165 patients who underwent RPN for localized cT1 renal tumors with warm ischemia at Kansai Medical University Hospital between August 2014 and December 2019 were retrospectively reviewed. Patients with multiple renal tumors or a solitary kidney were not included in this study. No patient underwent presurgical treatment with tyrosine kinase inhibitors or immune checkpoint inhibitors. All procedures were performed by experienced robotic surgeons at a single institution. Among these patients, two patients who underwent conversion to nephrectomy or open partial nephrectomy were excluded from the analysis. Ultimately, 163 patients were considered for further analyses.

### Data collection

The preoperative clinical data (sex, age, body mass index (BMI), American Society of Anesthesiologists (ASA) score, comorbidities of diabetes mellitus (DM) and hypertension (HTN), previous abdominal surgery, and antiplatelet or anticoagulant therapy), perioperative outcomes (WIT and estimated blood loss), and pathological features (pathological subtype and pathological T stage) were evaluated. Renal function was assessed by serum creatinine (SCr) and eGFR, which was calculated using the following equation established for the Japanese population^23^:

1 $$ {\text{eGFR}}\;\left( {{\text{mL}}/\min /1.73\;{\text{m}}^{2} } \right) = 194 \times {\text{Cr}}^{ - 1.094} \times {\text{age}}^{ - 0.287} \left( { \times 0.739\;{\text{for}}\;{\text{females}}} \right). $$

The percent reduction in renal function was calculated with the preoperative and postoperative (6 months after RPN) eGFRs. All patients underwent preoperative 3D-CT with or without contrast. Based on the imaging findings, nephrometry scoring systems including R.E.N.A.L., PADUA, and DAP scores were evaluated with several urologists at a preoperative conference. The lengths of the horizontal axis and vertical axis were measured at the transverse plane where the tumor area was the largest (x and y, respectively), and the length of maximal z axis was measured in the coronal or sagittal plane (z). Then, the tumor volume was calculated using the following equation applied to an ellipsoid (Supplementary Fig. 1):

2 $${\text{tumor}}\;{\text{volume}}\;\left( {{\text{cm}}^{3} } \right) = 4/3 \times \pi \left( {3.14} \right) \times x/2 \times y/2 \times z/2$$

The lengths of these three directions and the distance from the tumor to the collecting system were measured independently by two observers (HO and KA), each of whom was blinded to the clinical outcome.

### Instruments used in the study

R.E.N.A.L. score and DAP score were used in this study. The R factor in the R.E.N.A.L. system showed the maximal diameter of the tumor, with 1 point for a tumor size ≤ 4 cm and 2 points for a tumor size 4–7 cm. The E factor in the R.E.N.A.L. system showed the exophytic or endophytic properties of the tumor, with 1 point for ≥ 50% exophytic, 2 points for < 50% exophytic, and 3 points for entirely endophytic. The N factor in the R.E.N.A.L. system showed the nearness of the tumor to the collecting system, with 1 point for ≥ 7 mm, 2 points for 4–7 mm, and 3 points for ≤ 4 mm. The A factor in the R.E.N.A.L. system showed whether the tumor was located anterior (a) or posterior (p) to the kidney midline plane. When the tumor grew from the renal poles or arose from the kidney so that a meaningful anterior or posterior designation was not possible, (x) was assigned and no points were given. The L factor in the R.E.N.A.L. system showed the location relative to the polar lines, with 1 point indicating the lesion was above the upper or below the lower polar line, 2 points indicating the lesion crossed the polar line, and 3 points indicating > 50% of the mass was across the polar line, the tumor crossed the axial renal midline, or the mass was entirely between the polar lines. The D factor in the DAP system showed the maximal diameter of the tumor, with 1 point for a tumor size < 2.4 cm, 2 points for 2.4–4 cm, and 3 points for > 4 cm. The A factor in the DAP system showed the axial distance from the center point to the closest tumor edge, with 1 point for > 1.5 cm, 2 points for ≤ 1.5 cm, and 3 points for the tumor touching or overlapping the center point. The P factor in the DAP system showed the polar distance from the middle plane to the closest tumor edge, with 1 point for > 2 cm, 2 points for ≤ 2 cm, and 3 points for the tumor crossing of the axial renal midline.

### Statistical analysis

The primary outcome of this study was a reduced eGFR, which was defined as an eGFR reduction of ≥ 20% from baseline to 6 months after RPN. We used a clinically more significant cutoff point of 20% reduction of eGFR by referring to a previous report, wherein both 10% and 20% reduction of eGFR from baseline were used as cutoff values^11^. All continuous data are shown as median values and IQRs. The area under the receiver operating characteristic curve (AUC) was used to decide the cutoff value for continuous variables including tumor volume. Bivariate and multivariable logistic regression analyses were used to evaluate the relationship between clinical variables and reduced eGFR. A reduced model selection was performed using a backward step-down selection process in the multivariable analysis. The trend of our classification system for predicting changes in renal function and WIT was examined by performing a Jonckheere–Terpstra test. The abilities of our classification systems and previous nephrometry scoring systems to predict reduced eGFR were evaluated and compared using AUC analysis. The associations of the primary outcome with the clinical variables were measured by ORs and their corresponding 95% CIs. All statistical analyses were performed using EZR version 1.65 (Saitama Medical Center, Jichi, Japan)^24^. A two-sided p value < 0.05 was considered as statistically significant.

### Ethics approval

All procedures performed in the present study involving human participants were in accordance with the ethical standards of the institutional research committee and with the 1964 Helsinki Declaration and its later amendments or comparable ethical standards. The study was approved by the institutional review board of the Kansai Medical University Hospital, Japan (Approval No. 2020215), and informed consent was obtained from all individual patients prior to robotic partial nephrectomy.

## Supplementary Information

Supplementary Information.

## Data Availability

The datasets analysed during the current study are available from the corresponding author on reasonable request.

## Abbreviations

AML: Angiomyolipoma
ASA: American Society of Anesthesiologists
AUC: Area under the receiver operating characteristic curve
BMI: Body mass index
CI: Confidence interval
CKD: Chronic kidney disease
DAP: Diameter-axial-polar
DM: Diabetes mellitus
eGFR: Estimated glomerular filtration rate
HTN: Hypertension
IQR: Interquartile range
OR: Odds ratio
PADUA: Preoperative aspects and dimensions used for anatomic
PN: Partial nephrectomy
RCC: Renal cell carcinoma
R.E.N.A.L.: Radius, exophytic/endophytic, nearness to collecting system or sinus, anterior/posterior, and location relative to polar lines
RPN: Robotic partial nephrectomy
SCr: Serum creatinine
WIT: Warm ischemia time
3D-CT: Three-dimensional computed tomography
