# Supplementary material for: Tumor volume and tumor crossing of the axial renal midline predict renal function after robotic partial nephrectomy
Source: Sci Rep. 2021 Nov 18;11:22526. doi: 10.1038/s41598-021-01539-1 (PMC8602316; doi:10.1038/s41598-021-01539-1)

Supplementary Information

Tumor volume and tumor crossing of the axial renal midline predict renal function after robotic partial nephrectomy

Haruyuki Ohsugi, Kyojiro Akiyama, Hisanori Taniguchi, Masaaki Yanishi, Motohiko Sugi, Tadashi Matsuda, and Hidefumi Kinoshita

Department of Urology and Andrology, Kansai Medical University, 2-3-1 Shin-machi, Hirakata, Osaka 573-1191, Japan

Supplementary Table 1

New classification system for predicting postoperative renal function.

Variable	0 points	1 point
Tumor crossing of the axial renal midline	absent	present
Tumor volume, cm^3	<14.11	≥14.11

Supplementary Figure 1

a Representative three-dimensional computed tomography.
b Calculation of tumor volume (tumor volume (cm^3)=4/3×π(3.14)×x/2×y/2×z/2).

a

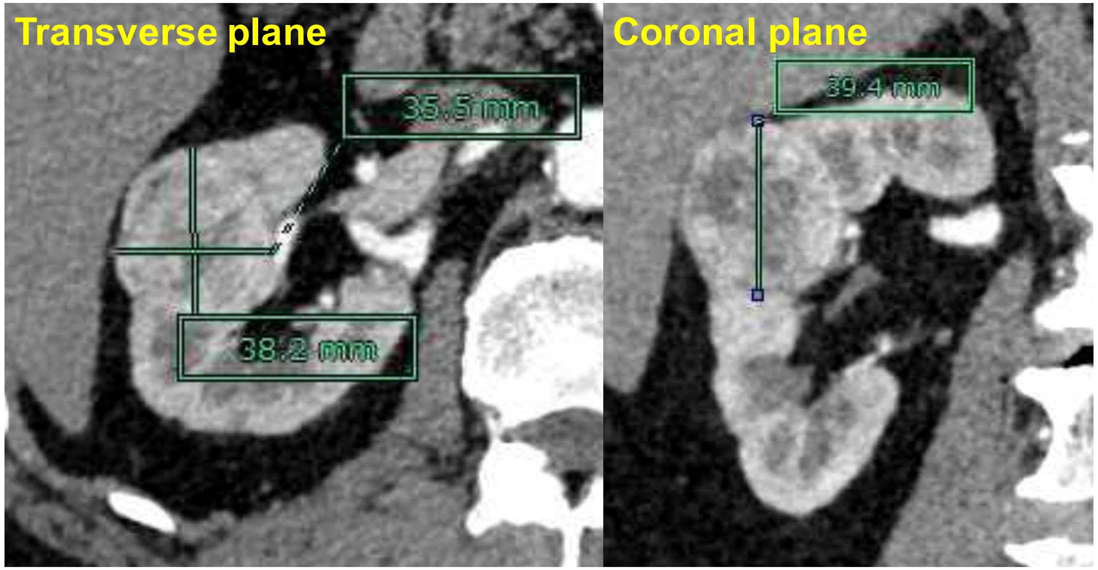

b

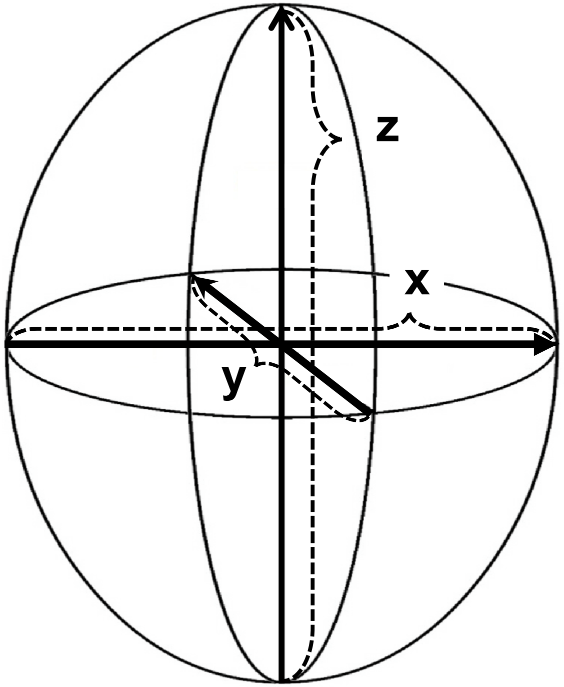

Supplementary Figure 2

Relationship between the distance from the tumor to the collecting system and **a** the P factor score of the DAP system or **b** tumor volume (R=0.481, p<0.001).
DAP diameter–axial–polar

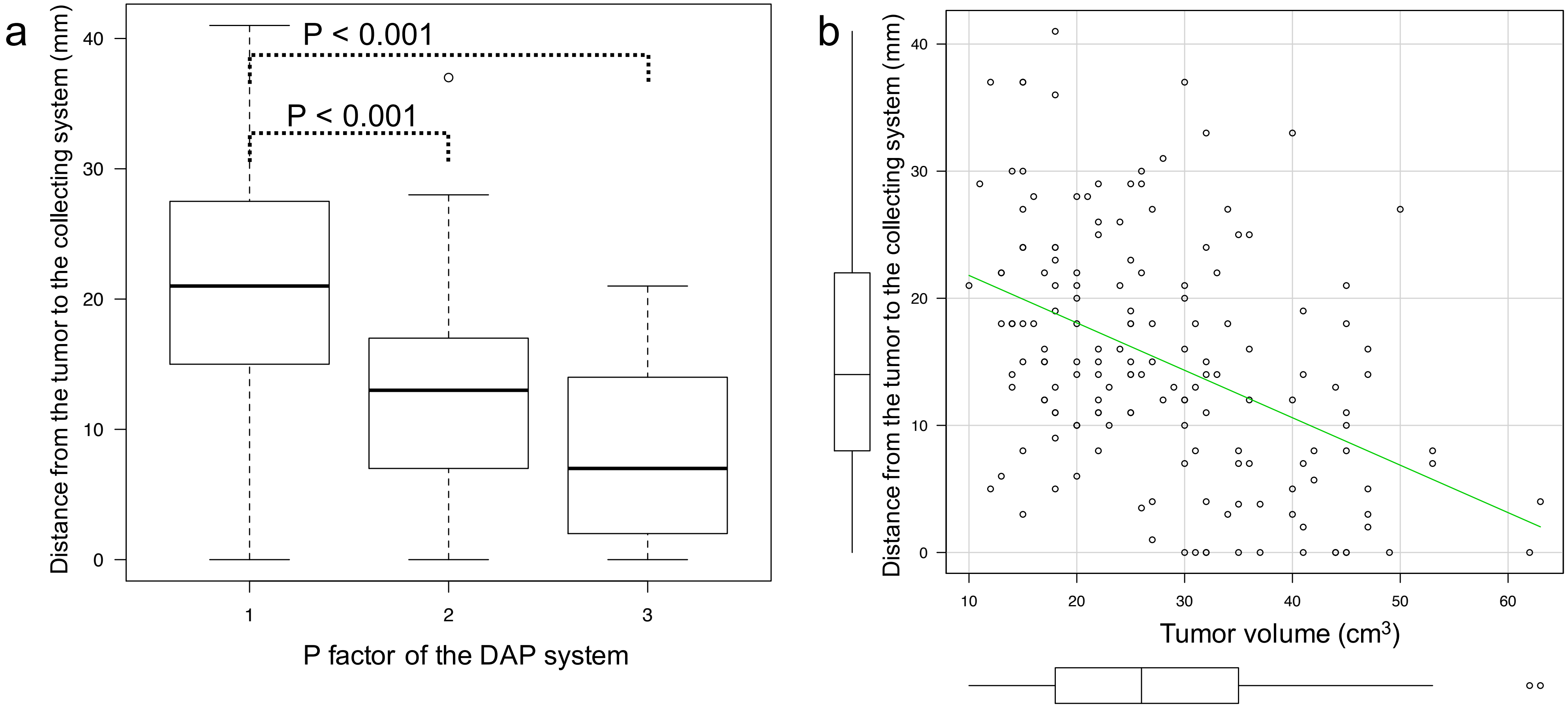

Supplement: Supplementary file 1 — Supplementary Information. [file 41598_2021_1539_MOESM1_ESM.pdf]
